# Thermophilic *Geobacillus* WSUCF1 Secretome for Saccharification of Ammonia Fiber Expansion and Extractive Ammonia Pretreated Corn Stover

**DOI:** 10.3389/fmicb.2022.844287

**Published:** 2022-05-25

**Authors:** Aditya Bhalla, Jessie Arce, Bryan Ubanwa, Gursharan Singh, Rajesh K. Sani, Venkatesh Balan

**Affiliations:** ^1^Department of Chemical and Biological Engineering, South Dakota School of Mines and Technology, Rapid City, SD, United States; ^2^Department of Chemistry, Biology and Health Science, South Dakota School of Mines and Technology, Rapid City, SD, United States; ^3^Great Lakes Bioenergy Center, Michigan State University, East Lansing, MI, United States; ^4^Department of Engineering Technology, College of Technology, University of Houston, Houston, TX, United States; ^5^Department of Medical Laboratory Sciences, Lovely Professional University, Phagwara, India

**Keywords:** *Geobacillus* sp., carbohydrate active enzymes, lignocellulose biomass, biofuels, biochemicals

## Abstract

A thermophilic *Geobacillus* bacterial strain, WSUCF1 contains different carbohydrate-active enzymes (CAZymes) capable of hydrolyzing hemicellulose in lignocellulosic biomass. We used proteomic, genomic, and bioinformatic tools, and genomic data to analyze the relative abundance of cellulolytic, hemicellulolytic, and lignin modifying enzymes present in the secretomes. Results showed that CAZyme profiles of secretomes varied based on the substrate type and complexity, composition, and pretreatment conditions. The enzyme activity of secretomes also changed depending on the substrate used. The secretomes were used in combination with commercial and purified enzymes to carry out saccharification of ammonia fiber expansion (AFEX)-pretreated corn stover and extractive ammonia (EA)-pretreated corn stover. When WSUCF1 bacterial secretome produced at different conditions was combined with a small percentage of commercial enzymes, we observed efficient saccharification of EA-CS, and the results were comparable to using a commercial enzyme cocktail (87% glucan and 70% xylan conversion). It also opens the possibility of producing CAZymes in a biorefinery using inexpensive substrates, such as AFEX-pretreated corn stover and Avicel, and eliminates expensive enzyme processing steps that are used in enzyme manufacturing. Implementing in-house enzyme production is expected to significantly reduce the cost of enzymes and biofuel processing cost.

## Introduction

Lignocellulose biomass is one of the most abundant renewable resources and is a sustainable alternative to fossil fuels to produce biofuel and biochemicals (da Costa Sousa et al., 2016a; Lopes et al., 2018; Kaur et al., 2020). Lignocellulose biomass is composed of three major constituents, namely cellulose, hemicellulose, and lignin. Pretreatment helps to disintegrate the complex network of lignin carbohydrate complex (LCC) in biomass to overcome biomass recalcitrance. Pretreated biomasses are subjected carbohydrate-active enzymes (CAZymes), such as glycosyl hydrolysate (GH), to produce fermentable sugars. These sugars are fermented using microorganisms to produce biofuel and biochemical (Jin et al., 2016; Østby et al., 2020; Sharma et al., 2020a; Singh and Arya, 2021). Researchers have taken several approaches to reduce the cost of enzymes needed to saccharify lignocellulosic Biomass. They include (i) bioprospecting and identifying new thermostable enzymes (Bouws et al., 2008; Couturier et al., 2012), (ii) incorporating novel enzyme-producing genes in the host organism to improve further the activity of the enzyme cocktail (Sharma et al., 2020b), (iii) improving the enzyme expression system by varying the promoter and regulatory elements (Drejer et al., 2018), (iv) evaluating synergistic interaction between different bacterial and fungal enzymes (Cortes-Tolalpa et al., 2017), and (v) producing enzymes in biorefinery using inexpensive substrates (Singh and Arya, 2019; Bajar et al., 2020).

When inoculated with lignocellulosic biomass, microorganisms such as fungi and bacteria secrete a suite of CAZymes, such as secretome, that de-construct complex substrate into fermentable monomeric sugars (Bhalla et al., 2013a). These sugars are used as a carbon source and are essential for the survival of organisms. The CAZymes in the secretome consist of different classes of enzymes such as GH, glycosyl transferases (GT), auxiliary activities (AA), carbohydrate esterase’s (CE), and polysaccharide lyases (PL). The most prominent CAZymes that are responsible for breaking down lignocellulosic biomass have cellulolytic (cellulose-degrading enzymes such as cellulase), hemicellulolytic (hemicellulose-degrading enzymes such as hemicellulase), ligninolytic (lignin-degrading enzymes such as laccase and peroxidase), and pectinolytic (pectin-degrading enzymes such as pectinase) activities (Adav et al., 2012a,b; Christopher et al., 2021; Guo et al., 2022). The composition of enzymes in the secretome is influenced by the different spatial and structural complexity of the different lignocellulosic biomasses’ components makeup (Grieco et al., 2020). Commercial enzymes are produced using an expensive substrate such as wheat bran and microcrystalline cellulose. Producing an enzyme cocktail in the biorefinery or using inexpensive feedstock such as native and pretreated lignocellulosic biomass will reduce enzyme production costs and greenhouse gas emissions (Johnson, 2016; Olofsson et al., 2017; Sun et al., 2018; Chettri et al., 2020).

It is difficult to identify individual hydrolyzing enzymes using routine enzyme assays in the laboratory, which only give a collective picture of the secretome’s enzyme activity. A proteomic analysis method based on liquid chromatography followed by tandem mass spectrometry (LC-MS/MS) has proven to be significant for profiling CAZymes in secretomes and acts as a powerful tool to identify enzymes that are otherwise impossible to detect (Bouws et al., 2008; Chundawat et al., 2011a). Furthermore, proteomic analysis of secretomes against genomic data using different bioinformatics tools provides more details about CAZymes and relative quantifications of enzymes in each sample. Various studies have reported determining the composition of secretomes on different untreated and pretreated lignocellulosic biomasses (Adav et al., 2012a; Arntzen et al., 2020; Thapa et al., 2020). However, studies about identifying CAZymes in secretome and comparing them with the hydrolytic potential of lignocellulosic biomass by carrying out hydrolysis and quantifying the sugars are scarce (Herrera et al., 2019; Filiatrault-Chastel et al., 2021). Having a good knowledge about enzyme composition in secretomes and correlating it with lignocellulosic biomass sugar conversion gives a comprehensive insight into the repertoire of enzymes required for the efficient conversion of lignocellulosic biomass in a biorefinery (Özdenkçi et al., 2017; Sethupathy et al., 2021).

*Geobacillus* strain WSUCF1 is a gram-positive, rod-shaped, aerobic or facultatively anaerobic, and a spore-forming thermophilic bacterium that secretes thermostable cellulases, hemicellulases, and laccases when grown on lignocellulosic substrates (Rastogi et al., 2010). The genome of this microbe was also sequenced and annotated before (Bhalla et al., 2013b). We have re-annotated the genome and identified some novel enzymes not identified before. Subsequently, we produced secretomes using different substrates, such as Avicel (AVI), xylan (XYL), untreated corn stover (UT-CS), ammonia fiber expansion-pretreated corn stover (AFEX-CS), and extractive ammonia-pretreated corn stover (EA-CS), that removes about 44% of lignin for the first time using established methods (Balan et al., 2009; da Costa Sousa et al., 2016a). The respective secretomes produced are labeled (AVI-S, XYL-S, UTC-S, AFC-S, and EAC-S) using the substrates mentioned above. The respective secretomes were subjected to proteomic analysis using established protocols (Chundawat et al., 2011b). Two secretomes (AVI-S and AFC-S) that gave the highest enzyme activities were used to hydrolyze AFEX-CS and EA-CS by combining with commercial enzymes at different ratios. Since WSUCF1 bacterial secretomes lack exo-cellulase activities, we doped the secretomes with purified fungal cellobiohydrolase I and II enzymes (CBHI and CBHII). Since the whole fermentation broth can be used directly for enzymatic hydrolysis, this will help to reduce the cost associated with adding stabilizers, formulation, concentration, refrigeration, and transportation by enzyme companies as reported before (Humbird et al., 2011; Culbertson et al., 2013; Dragone et al., 2020; Saini and Sharma, 2021). Our pretreated biomass hydrolysis study has confirmed the synergy operating between *Geobacillus* WSUCF1 bacterial and commercial fungal enzymes. Adding a small portion of commercial enzymes to compensate for the missing cellobiohydrolase I and II enzyme activities in bacterial secretomes will produce sugar yield comparable to using commercial enzymes alone in a biorefinery. This approach is expected to reduce the overall biofuel and biochemical production cost.

## Materials and Methods

### ERGO Annotations

Protein annotations were obtained from the ERGO genome analysis suite (Zhang et al., 2018) using the protocol presented in previous publications (Overbeek et al., 2003).

### Lignocellulosic Biomass and Chemical Source

The pre-milled CS used was obtained from the Great Lakes Bioenergy Center (GLBRC). The stover was produced from corn seeds (Pioneer 36H56). We harvested the corn stover in Wisconsin in November 2009 and stored it in zip-lock bags with an 8% moisture content at room temperature. All the research chemicals, buffer salt, and fermentation media used in these studies were purchased from Sigma–Aldrich.

### Ammonia Fiber Expansion and Extractive Ammonia Pretreatment

A high-pressure 5-gallon 365 stainless steel PARR reactor, Parr Instrument Company, IL was used to produce AFEX-CS. The reaction was carried out in a walk-in hood under the following parameters: 1:1 (weight/weight) ratio of ammonia to biomass with 60% moisture content for 30 min at 100^°^C (Balan et al., 2009; Chundawat et al., 2020). After completing the AFEX processes, the pretreated samples were kept in a hood overnight on a plastic tray to remove any residual ammonia bound to the biomass. The EA pretreatment was carried out using the tubular reactor-like protocol reported in the literature (da Costa Sousa et al., 2016b). Briefly, the pretreatment was carried out in a high-pressure stainless-steel reactor vessel at 121^°^C for 30 min under the following pretreatment conditions: 3:1 (weight/weight) ammonia to biomass ratio with a 10% moisture content. To determine the composition of both the untreated and the pretreated biomass, NREL protocols were utilized (Sluiter et al., 2010). Since AFEX is a dry-to-dry process, untreated and pretreated CS compositions were similar (Glucan 35.7%, xylan 23.9%, lignin 17.4%, and others 23%). This pretreatment process removed 16 wt% of the biomass, which is about 44 wt% of the total lignin available in the biomass. The composition of EA-CS was found to be glucan 32.4%, xylan 23.5%, lignin 12.2%, and others 15.7%.

### Commercial Enzymes

Three commercial enzyme mixtures were used in these experiments. The first two enzyme cocktails Cellic^®^ CTec2 (138 mg protein/ml, batch number VCNI 0001), a complex blend of cellulase, β-glucosidase, and hemicellulase and Cellic^®^ HTec2 (157 mg protein/ml, batch number VHN00001) were generously provided by Novozymes (Franklinton, NC, United States). The third enzyme cocktail Multifect Pectinase^®^ (72 mg protein/ml, batch number 4861295753) was a gift from DuPont Industrial Biosciences (Palo Alto, CA, United States). The protein concentrations of the enzymes were determined by estimating the protein (and subtracting the non-protein nitrogen contribution) using the Kjeldahl nitrogen analysis method (AOAC Method 2001.11, Dairy One Cooperative Inc., Ithaca, NY, United States). Exocellulases CBH I and CBH II used in this study were purified from a commercial enzyme source. The purification and expression methodologies and enzyme characteristics have been described previously using a combination of ion-exchange and size-exclusion chromatography (Gao et al., 2010a).

### Producing Ammonia Fiber Expansion-Pretreated Corn Stover Hydrolysate

The AFEX-CS was hydrolyzed at 6% glucan loading using Ctec2 and Htec2 enzymes based on the reported procedure (Jin et al., 2016). The hydrolyzed samples were heat-treated at 100°C for 10 min using a hot plate and centrifuged to remove unhydrolzyed solids and precipitated protein. The supernatant was subjected to 0.2 mm nylon syringe filer and the resulting AFEX-CS hydrolyzate was stored in a −20^°^C freezer until they were used as substrates to grow *Geobacillus* sp. WSUCF1 for producing secretome.

### Microorganism, Culture Conditions, and Secretome Production

The strain used in this study, *Geobacillus* WSUCF1 gram-positive, rod-shaped, aerobic or facultatively anaerobic, was isolated from soil samples provided by a compost facility at Washington State University, Pullman, WA. The WSUCF1 strain was grown on a minimal media with a pH of 7.0 at 60^°^C. Pure substrates (xylan or Avicel-0.2%) and lignocellulosic substrates (AFEX-CS, EA-CS or UT-CS, or AFEX-CS hydrolyzate, 0.5%) were used to supplement the minimal medium as the carbon and energy source, respectively. The control experiment consisted of a carbon source without inoculum. The minimal medium was prepared as follows per liter: 0.1 g MgSO_4_.7H_2_O; 0.2 g yeast extract; 0.1 g nitrilotriacetic acid; 0.05 g CaCl_2_.2H_2_O; 0.010 g casamino acid; 1-ml FeCl_3_ solution (0.03%); 1.8 g of 85% H_3_PO_4_; 0.005 g methionine; 0.01 g NaCl; 0.3 g NH_4_Cl; 0.01 g KCl; and 1-ml of Nitsch’s trace elements (Rastogi et al., 2010). To inoculate 400 ml of the minimal medium, 20 ml of pre-grown culture for 24 h was used. The 400 ml minimal medium contained the same substrates used in the 2,000 ml inoculum in the Erlenmeyer flasks. The flasks were placed in an Eppendorf Innova 44R shaker incubator under the following parameters: 60*^o^*C, 150 rpm for a duration of 96 h in a control flask without inoculum, and only carbon sources were used for each experiment under similar conditions. Immediately after producing the secretome, the samples were centrifuged using Beckman Coulter Centrifuge Avavti J-20XP at 4^°^C and 10,000 *g* for 10 min. Supernatants containing the secretomes were analyzed for xylanase and cellulase activity using a model substrate, such as para nitrophenol (p-NP) linked sugars, and substrates such as xylan, Avicel, and CMC using reported procedures.

### Concentrating Secretome and Estimating Protein Concentration

The supernatant containing the enzymes was filtered using a 0.2-μm membrane filter and concentrated using 10 kDa cut-off membrane cassettes. The concentrated enzymes were stored at 4°C in a refrigerator or stored in 10% glycerol stock at −80°C for long-time storage. Protein concentration was estimated using a 2D-quant kit acquired from GE healthcare life Sciences or using the bovine serum albumin (BSA) assay kit.

### Secretome Activity Assays

The complex substrates carboxymethyl cellulose (CMC; Sigma), birchwood xylan (Sigma), and Avicel PH101 (Fluka) were used for the assay of exoglucanase, xylanase, and Avicelase activities, respectively (Gao et al., 2010a,b, 2011). The reaction mixtures contained 200 μl of the diluted enzyme with 200 μl of 2% (weight/volume) substrate in 100 mM phosphate buffer or 100 mM citric buffer depending on the enzyme. For xylanase activity analysis, we used 100 mM phosphate buffer at pH 6.5, and for cellulase we used 100 mM citric buffer at pH 5.0. The enzyme–substrate mixture reaction was heated at 70°C for different intervals of time, and the addition of 600 μl of 3,5-dinitrosalycic acid (DNS) was used to stop the reaction. Subsequently, the mixture was heated for 10 min and brought to room temperature by placing on ice for color stabilization. The samples were analyzed at 540 nm and xylose (reducing sugars) was measured against a xylose standard curve. From the calculations, one unit of xylanase was defined as the amount of enzyme needed to release 1 μmol of xylose. The same conditions were given to the other enzymes used; one unit of endoglucanase equaled the amount of enzyme needed to release 1 μmol of glucose equivalent from carboxymethyl cellulose/min. One unit of Avicelase was calculated as the amount of enzyme needed to release 1 μmol of glucose equivalent from Avicel/min.

### Protein Identification Using LC-MS/MS Analysis

Sodium dodecyl sulfate-polyacrylamide gel electrophoresis (SDS-PAGE) was used on 50–100 μg of protein in each sample at 100 V for 10 min. Sample lanes were single-handedly cut into small pieces. The gel used was stored in 10% acetic acid until analyzed. Following Shevchenko method with a few modifications, gel bands were digested in-gel (Shevchenko et al., 2006). To summarize, 100% acetonitrile was used to dehydrate SDS-PAGE gel pieces. Then, they were incubated for 45 min at 56°C under the following conditions: with 10 mM dithiothreitol in 10 mM ammonium bicarbonate at pH 8. After 45 min of incubation, the sample was dehydrated for the second time and incubated using 50 mM iodoacetamide in 100 mM NH_4_HCO_3_ for 20 min in a completely dark setting. After 20 min of incubation, the samples (gel bands) were taken out and washed with ammonium bicarbonate and dehydrated for the third time. In 50 mM NH_4_HCO_3_, 0.01 μg/μl of sequencing grade modified trypsin was prepared. About 50 μl of the trypsin solution prepared was added to each gel piece until it was submerged, and the resulting bands were incubated overnight at 37°C. Sonication was then used to extract peptides from the gel in a water bath solution of 60% acetonitrile, 1% tricarboxylic acid, and vacuum dried to ∼2 μl using Eppendorf vacufuge.

The peptides extracted previously were resuspended in a 2% acetonitrile and 0.1% tricarboxylic acid to a total of 20 μl. Waters nanoAcquity sample manager automatically injected 10 L of re-suspended peptides into a Waters Symmetry C18 peptide trap (5 mm, 180 mm × 20 mm) and loaded it for 5 min in 2% ACN/0.1% formic acid. By using the Waters nanoAcquity UPLCP (buffer A = 99.9% water, 0.1% formic acid, buffer B = 99.9% acetonitrile, 0.1% formic Acid), the bound peptides were eluted onto a Waters BEH130 C18 column (0.150 mm × 100 mm, 1.7 mm) and eluted over 90 min. In 77 min, at a flow rate of 1 L/min, a gradient of 5% B to 30% B was achieved.

Michrom ADVANCE nano spray source was used to spray eluted peptides into a Thermo Fisher LTQ-FT Ultra mass spectrometer.^1^ FT survey scans were taken (25,000 resolution at *m/z* 400), and for each survey scan, in the LTQ, the top 10 ions were subjected to collision-induced dissociation at a low energy. In BioWorks Browser v3.3.1 (Thermo Fisher Scientific), the resulting MS/MS spectra are converted in to peak lists and searched against all bacterial proteins in NCBI using the default parameters. The results were also compared with two custom databases consisting of *Geobacillus* sp. By using the Mascot search algorithm, v2.4,^2^ we searched for ORFs or translated protein sequences appended with common lab contaminants. In addition, Mascot output was analyzed using the Scaffold, v4.0.3 software tool^3^ using Protein Prophet computer algorithm to probabilistically validate the identification of proteins (Kaur et al., 2020). Assignments with validations above the scaffold’s 95% confidence level are considered valid.

### Enzymatic Hydrolysis of Ammonia Fiber Expansion-Pretreated Corn Stover and Extractive Ammonia-Pretreated Corn Stover

Hydrolytic capabilities of secretomes AVI-S and AFC-S were tested utilizing AFEX-CS and EA-CS as substrates. Following a high-throughput method for enzymatic hydrolysis (Chundawat et al., 2008), 0.5 ml reaction volume was used with glucan loaded at 0.2% (∼0.6% solids loading). For hydrolysis, WSUCF1 strain secretomes, commercial enzyme and combination mixture, and a combination of WSUCF1 strain secretomes and purified exoglycanase CBHI and CBHII were studied at two protein loadings, namely 15 and 60 mg/g glucan. A tumbling reactor was used to carry out the hydrolysis reaction at 55°C and pH 5.0 for 24 h. The sugar released after enzyme hydrolysis for some of the samples were estimated using high performance liquid chromatography using Biorad Aminex HPX-87P column against the respective standards at 60°C using water as the mobile phase. For majority of the samples enzyme-based glucose analysis kits (R-Biopharm, Marshall, MI) and xylose analysis kits (Megazyme, Bray, Ireland) were used. We define synergy operating between the enzyme during hydrolysis when the sugar yield was higher while adding enzyme mixture to the sum of the sugar yield achieved with respective enzymes when used separately in the same amounts as in the mixture.

### Annotating Glycoside Hydrolase Families

Glycoside hydrolases in the WSUCF1 strain genome were predicted using dbCAN2 (Zhang et al., 2018). The ERGO predicted protein set was submitted to the dbCAN2 Meta server. To annotate for GH enzymes, dbCAN2 used three tools to search for CAZyme and pre-annotated CAZyme sequence databases. They include HMMER and DIAMOND, and CAZyme short peptides were searched using Hotpep (Lombard et al., 2014; Munir et al., 2014; Hüttner et al., 2017). In the WSUCF1 strain, 118 CAZymes were predicted, and of these 44 were labeled under a GH family. Gene hits that had at least two of the three tools predicting the same GH family were kept. After filtering, 34 GH enzymes were remaining.

### Phylogenetic Tree Construction

Laccase, endo-1,2-β-xylanase, and endoglucanase M protein sequences were submitted to NCBI-BLAST to gather similar sequences to construct phylogenetic trees. BLAST hits with e-values < 1e^–16^ were selected. MUSCLE was used to create multiple sequence alignment with gathered sequences from BLAST searches. Then the phylogenetic trees were constructed with PhyML (Guindon et al., 2010) through their web-based application. The substitution model used was Bayesian information criterion (BIC), the nearest neighbor interchange was used for tree searching, and aBayes was used for branch support.

## Results and Discussion

### Glycoside Hydrolases Found in *Geobacillus* WSUCF1

**FIGURE 1:**
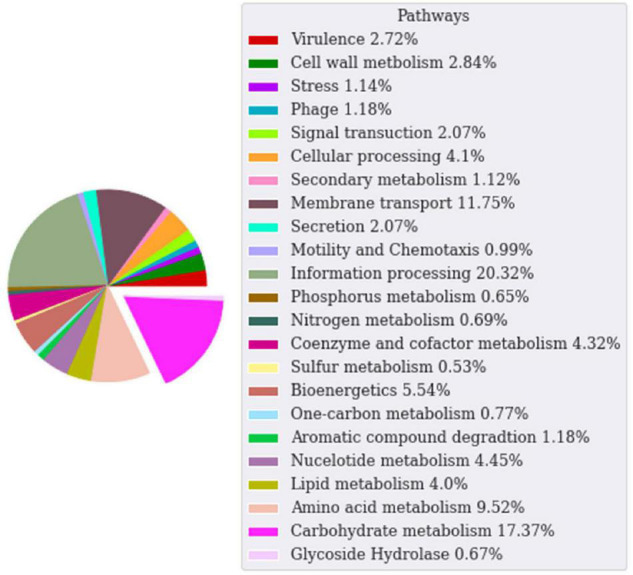
Pie chart of ERGO annotations for *Geobacillus* sp. WSUCF1 bacterial strain categorized based on the pathway they participate in. Each wedge in the pie chart represents the percentage of annotations for that pathway. The carbohydrate and glycoside hydrolase enzymes are grouped together.

The genome sequence of the WSUCF1 strain is 3.4 Mb. Genome annotation was done via the ERGO Suite (Bhalla et al., 2013b). Subsequently, we used the dbCAN2 meta server, which contains three tools DIAMOND, HAMMER, and Hotpep, to search for CAZymes. Then we filtered the data to identify specific GH families. In the ERGO annotations, 17.8% of the *Geobacillus* WSUCF1 genes are responsible for carbohydrate metabolism and 0.67% are GH enzymes (Figure 1). Cellulases, xylanases, and laccases from the WSUCF1 strain have been reported in the literature (Rastogi et al., 2010; Bhalla et al., 2013a; Rai et al., 2019). Enhanced hydrolysis of lignocellulosic biomass was reported with the doping of a thermostable recombinant laccase produced using genes isolated from WSUCF1 strain (Rai et al., 2019; Govil et al., 2020). Thus, on the exploration of the genome sequence of WSUCF1, we could identify several new genes encoding GH enzymes, which makes it a potential candidate for lignocellulose biomass conversion. The genome of WSUCF1 in total showed 34 GH enzymes with 19 different GH families. The WSUCF1 strain was found to contain 7 genes from the GH13 family, 4 genes from GH1 and GH43 family, 3 genes from the GH18 family, 2 genes from the GH51 family, and 1 from GH36, GH42, GH32, GH27, GH67, GH23, GH127, GH10, GH4, GH25, GH2, GH52, GH39, and GH130 family, respectively. Table 1 shows the comparison of GH enzyme numbers in WSUCF1 to other CAZyme producing organisms reported in the literature.

**TABLE 1:** Count comparisons of GH enzymes in *Geobacillus* sp. *strain* WSUCF1 to other organisms.

Biomass degrading bacterial and fungal species	Number of GH enzyme	References
*Penicillium subrubescens*	410	Herrera et al., 2019
*Penicillium rubens*	222	Herrera et al., 2019
*Penicillium chrysogenum*	234	Herrera et al., 2019
*Talaromyces stipitatus*	271	Herrera et al., 2019
*Aspergillus niger*	252	Herrera et al., 2019
*Aspergillus oryzae*	304	Herrera et al., 2019
*Aspergillus nidulans*	275	Herrera et al., 2019
*Trichoderma reesei*	200	Herrera et al., 2019
*Geobacillus* sp. strain WSUCF1	34	This work
*Geobacillus thermodenitrificans* T12	33	Thapa et al., 2020
*Geobacillus stearothermophilus* DSM 458	17	Thapa et al., 2020
*Clostridium termitidis*	199	Herrera et al., 2019
*Bacillus licheniformis* 0DA23-1	60	Thapa et al., 2020
*Pseudomonas putida*	30	Thapa et al., 2020
*Streptomyces actuosus* ATCC 25421	161	Thapa et al., 2020

### Lignocellulolytic Enzymes Found in WSUCF1

**FIGURE 2:**
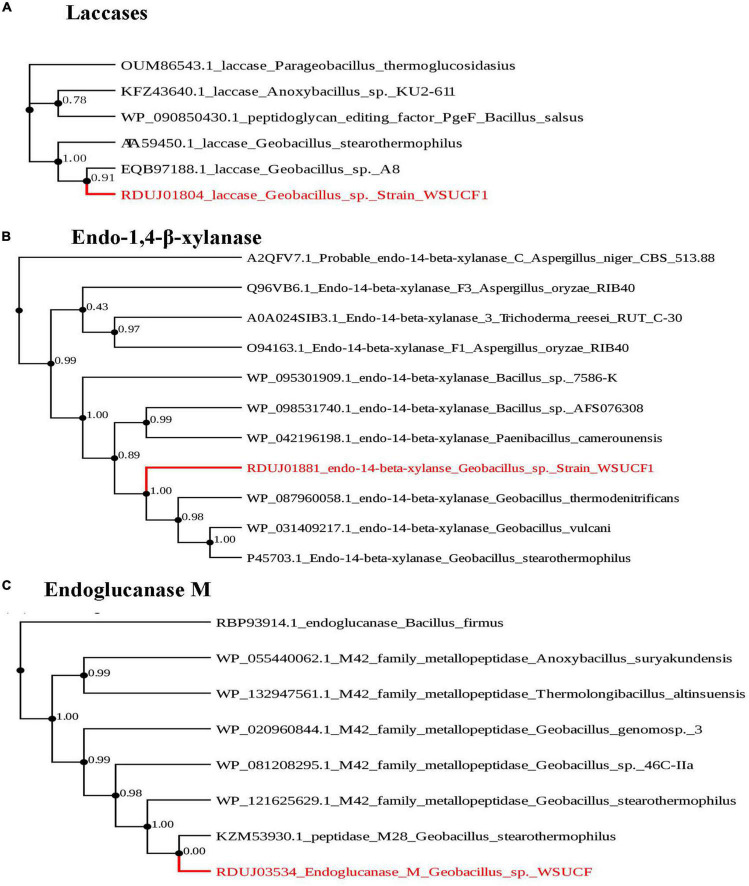
Phylogenetic tree of key enzymes found in *Geobacillus* sp. *strain WSUCF1.* Here, **(A)** phylogenetic tree of laccase from *Geobacillus* sp. *strain WSUCF1* and other laccases from *Parageobacillus thermoglucosidasius*, *Anoxybacillus* sp. *KU2-611*, *Bacillussalsus*, *Geobacillus stearothermophilus*, *and Geobacillus stearothermophilus.* Laccase from *Geobacillus* sp. *strain WSUCF1* was identified with HAMMER. **(B)** Phylogenetic tree of endo-1,4-β-xylanase from G*eobacillus* sp. *strain WSUCF1* and other endo-1,4-β-xylanases from fungi *Trichoderma reesi* RUT C-30, *Aspergillus oryzae* RIB40, *Aspergillus Niger* CBS 513.88 and bacteria *Paenibacillus camerounensis, Bacillus* sp. AFS0763308, *Geobacillus vulcani, Geobacillus stearothermophilus*, *Geobacillus thermodenitrificans*, and *Bacillus* sp. 7586-k. **(C)** Phylogenetic tree of Endoglucanase M from G*eobacillus* sp. *strain WSUCF1* and one endoglucanase from *Bacillus firmus* and peptidases from *Anoxybacillus suryakundensis*, *Thermologi Bacillus altinsuensis*, *Geobacillus genome* sp. *3*, *Geobacillus* sp. *46C-IIa*, and *Geobacillus stearothermophilus*. The nearest neighbor interchange was used for tree searching, and aBayes was used for branch support. PhyML was used to create the tree which uses Maximum likelihood methods. The substitution model used was Bayesian information criterion (BIC), nearest neighbor interchange was used for tree searching, and aBayes was used for branch support.

The WSUCF1 bacterial genome contains several hemicellulases, a few cellulases, and a few lignin-degrading enzymes. Among the 17 types of hemicellulases that were found, two were endo-1,2-β-xylanases, four were β-xylosidases, two were α-L-arabinofuranosidases, and one each of arabinan endo-1,2-α-L-arabinosidase, α-galactosidase, TREHALOSE-6-phosphate hydrolase, 1,2-α-glucan branching enzyme, exo-α-1,2-glucosidase, pullulanase, α-amylase, neopullulanase, and xylan α-1,2-glucuronidase. Only two types of cellulases were found, and they included two endoglucanase M and a 6-phospho-β-glucosidase. About five lignin-degrading enzymes were found, and they included two thioredoxin peroxidases, two peroxidases, namely cytochrome c peroxidase and glutathione peroxidase, and a laccase (Table 2). Phylogenetic trees were constructed for three lignocellulolytic enzymes (Figure 2). Those enzymes were laccase, endo-1,2-β-xylanase, and endoglucanase M. The laccase identified in this study is identical to the laccase reported in the literature before we confirmed using pairwise alignment (Rai et al., 2019).

**TABLE 2:** List of Hemicellulases, Cellulases, and Laccases/Peroxidases in *Geobacillus* sp. WSUCF1 bacterial strain.

Accession	GH family and AA family	Description	EC number
**Hemicellulases**			
RDUJ01881	GH10	Endo-1,2-β-xylanase	EC 3.2.1.8
RDUJ00171	GH13	Pullulanase	EC 3.2.1.41
RDUJ00324	GH13	α-amylase	EC 3.2.1.1
RDUJ01367	GH13	Trehalose-6-phosphate hydrolase	EC 3.2.1.93
RDUJ03669	GH13	1,2-α-glucan branching enzyme	EC 2.4.1.18
RDUJ03692	GH13	Exo-α-1,2-glucosidase	EC 3.2.1.20
RDUJ03802	GH13	Neopullulanase	EC 3.2.1.135
RDUJ04219	GH13	1,2-α-glucan branching enzyme	EC 2.4.1.18
RDUJ03638	GH36	α-galactosidase	EC 3.2.1.22
RDUJ01887	GH39	β-xylosidase	EC 3.2.1.37
RDUJ00503	GH43	β-xylosidase	EC 3.2.1.37
RDUJ00980	GH43	Arabinan endo-1,2-α-L-arabinosidase	EC 3.2.1.99
RDUJ03916	GH43	Endo-1,2-β-xylanase	EC 3.2.1.8
RDUJ04450	GH43	β-xylosidase	EC 3.2.1.37
RDUJ03917	GH51	α-L-arabinofuranosidase	EC 3.2.1.55
RDUJ03919	GH51	α-L-arabinofuranosidase	EC 3.2.1.55
RDUJ01879	GH52	β-xylosidase	EC 3.2.1.37
RDUJ01886	GH67	Xylan α-1,2-glucuronidase	EC 3.2.1.131
**Cellulase**			
RDUJ02447	GH4	6-phospho-β-glucosidase	EC 3.2.1.86
RDUJ03534	GH9	Endoglucanase M	EC 3.2.1.4
RDUJ00179	GH9	Endoglucanase M	EC 3.2.1.4
**Laccases/Peroxidases**			
RDUJ02824	AA2	Thioredoxin peroxidase	EC 1.11.1.15
RDUJ02895	AA2	Glutathione peroxidase	EC 1.11.1.15
RDUJ01427	AA2	Thioredoxin peroxidase	EC 1.11.1.15
RDUJ00922	AA2	Peroxidase	EC 1.11.1.7
RDUJ00921	AA2	Peroxidase	EC 1.11.1.7
RDUJ02184	AA2	Cytochrome c peroxidase	EC 1.11.1.5
RDUJ01804	AA1	Laccase	EC 1.10.3.2

*Here, AA, auxiliary activity; GH, Glycosylic hydrolase.*

Additionally, laccase clustered very well with other laccases from other *Geobacillus* sp., and endo-1,2-β-xylanase clustered with endo-1,2-β-xylanases from other *Geobacillus* sp. Additionally, the endo-1,2-β-xylanases from the WSUCF1 strain show a distant relationship with endo-1,2-β-xylanase from fungal species *Trichoderma reesei* and *Aspergillus oryzae*. Endoglucanase M was included in the phylogenetic analysis to verify its identity with different endoglucanase reported in the literature. It is interesting to note that WSUCF1 strain endoglucanase-M clustered very well with peptidases from other *Geobacillus* sp.

### Producing Secretomes Using Different Substrates

**FIGURE 3:**
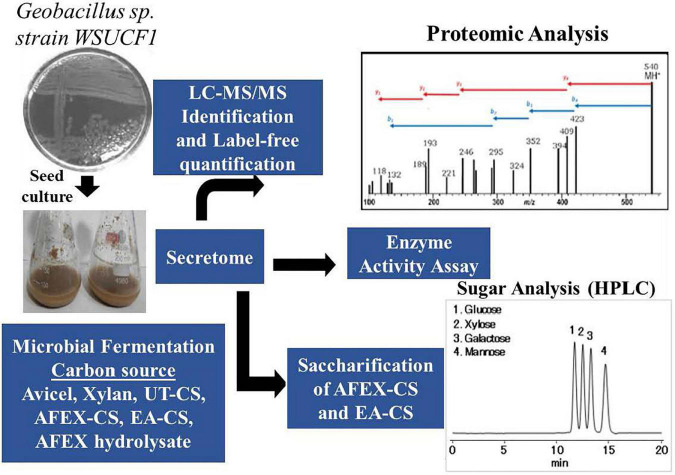
Picture showing how secretome was produced using different substrates and carbon sources and used for proteomic analysis, enzyme activity assay, and saccharification of AFEX-CS and EA-CS.

*Geobacillus* sp. strain WSUCF1 single-colony culture was tooth-picked from LB agar plate and used to produce the seed culture in growth media in a shake flask. An appropriate amount of seed culture was inoculated to different lignocellulosic substrates (UT-CS, AFEX-CS, and EA-CS), AFEX-CS hydrolysate and natural substrates (xylan, Avicel) as carbon sources to produce secretomes. These secretomes were centrifuged and further passed through a 0.2-mm filter and concentrated using molecular weight cut-off centrifugal concentrators and stored in the refrigerator at 4°C or stored in 10% glycerol stock at -80°C in the freezer (Lau et al., 2012). Some of the concentrated secretome enzymes were used for different analyses such as (i) proteomics using LC-MS/MS, (ii) activity assay using pNP bound to sugars and native substrates (Avicel or xylan or CMC), and (iii) saccharification experiments using AFEX-CS and EA-CS as substrates with commercial and purified enzymes (Figure 3). The fermentable sugars (glucose and xylose) produced after enzyme hydrolysis were analyzed using HPLC or sugar assay kit to determine their concentration.

### Enzyme Activity Assays

*Geobacillus* WSUCF1 strain when cultured using different substrates, such as UT-CS, AFEX-CS, EA-CS, Avicel, Xylan, and hydrolysate produced respective secretomes (UTC-S, AFC-S, EAC-S, AVI-S, XYL-S, and HYL-S). The secretomes were found to contain a varying concentration of enzymes. To determine the hydrolytic capacity of all six secretomes, we adopted an existing high-throughput microplate method to quantify enzyme activity in microplates (Chundawat et al., 2008). Briefly, 250 μl of 2.5% (w/v) stock substrate was added to a 2.2 ml deep-well microplate (Greiner, Monroe, NC). The substrates were Avicel, oat spelt xylan, and CMC. About 50 μl of 0.5 M citrate buffer with pH of 4.5 was added along with 200 μl of the desired diluted desalted enzymes (20 ng to 1,000 μg/well). To avoid interference with enzyme activity and reducing sugar assays, an Hi-Prep 26/10 desalting column (GE Healthcare, Buckinghamshire, United Kingdom) was used to desalt all the enzyme preparations. A 2D-quant or BSA assay kit was used to estimate the protein concentration in desalted fractions. The microplates were incubated at 50°C for 60 min while shaking continuously at 250 rpm. The reagent used to estimate the total reducing sugars released was 3,5-dinitrosalicylic acid (DNS). One unit of CMCase, Avicelase, and xylanase activity was defined as 1 μM of reducing sugars (as glucose equivalents) released per milligram of enzyme per minute. CMCase activity of different secretomes was found to be in the following decreasing order: AVI-S > AFC-S > EAC-S > UTC-S > XYL-S. Xylanase activity of different secretomes were found to be in the following decreasing order EAC-S > XYL-S > UTC-S > AFC-S > AVI-S. Avicelase activity of different secretomes were found to be in the following decreasing order AVI-S > AFC-S > EAC-S > UTC-S > XYL-S (Table 3).

**TABLE 3:** Secretome enzyme activity assay results.

Secretomes	Relative enzyme activities*		
	Xylanase	CMCase	Avicelase
XYL-S	94.3	11.8	6.5
AVI-S	70.0	100.0	100.0
UTC-S	87.4	30.6	7.8
AFC-S	78.7	58.4	10.4
EAC-S	100.0	37.8	8.3

*Activity assays were carried out three times and the results are within 0.5% error range.

### Proteomic Analysis of Different WSUCF1 Secretome

To understand how WSUCF1 secretomes are composed, LC-MS/MS analysis was carried out on different secretomes and fragmented peptide traces were obtained. A fully automatic ThermoScientific HF-X mass spectrometer was used to analyze the samples. For initial identification of the unknown proteins, data were searched against the SwissProt, UniProtKB, and NCBI protein database for bacterial and fungal species. A forward and reversed database search was performed. In the process of obtaining quantitative spectral counts, top hits were identified for each of the proteins, which were then used in the search for obtaining spectral counts. Each sample was analyzed in quadruplicate, and standard deviations per sample were less than 10%. Data showed the presence of endo-1,2-β-xylanase, β-xylosidase, α-L-arabinofuranosidases, arabinan endo-1,2-α-L-arabinosidase, α-galactosidase, trehalose-6-phosphate hydrolase, pullulanase, neopullulanase, xylan α-1,2-glucuronidase, endoglucanase M, 6-phospho-β-glucosidase, thioredoxin peroxidases, peroxidase, or glutathione peroxidase depending on the substrate enzyme composition of the secretomes varied.

**FIGURE 4:**
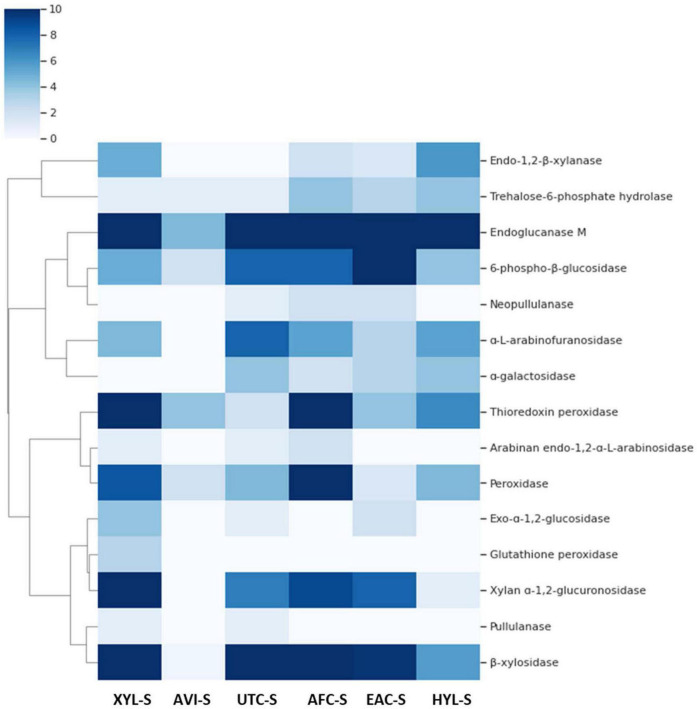
Heat map of spectral counts of different enzymes such as cellulases, hemicellulases, and peroxidases present in secretomes of *Geobacillus* sp. WSUCF1 bacterial strain. Each row represents an enzyme, and each column represents secretome producing using different substrates given in bracket: XYL-S (Xylan), AVI-S (Avicel), UTC-S (UT-CS), AFC-S (AFEX-CS), EAC-S (EA-CS), and HYL-S (AFEX-CS Hydrolysate). The cells with the darker turquoise color had the higher spectral count showing a higher concentration of enzymes and lightly colored cells had a lower spectral count with lower enzyme concentration. The dendrogram on the left side of the heatmap clusters the enzymes that have similar spectral counts across all substrates.

**FIGURE 5:**
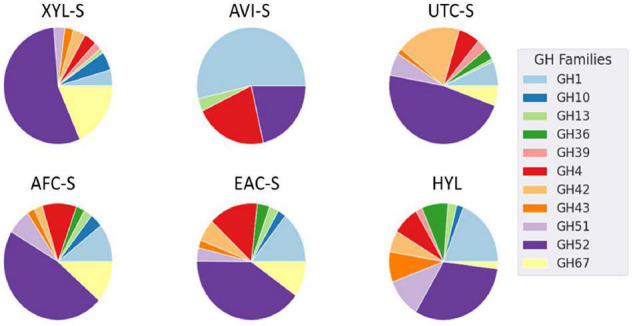
Pie chart of spectral counts from *Geobacillus* sp. WSUCF1 secretomes grown on different substrates. Only GH enzymes annotation were shown in the figure. Here, each pie chart represents a secretome and the corresponding substrates used are given in the bracket XYL-S (Xylan), AVI-S (Avicel), UTC-S (UT-CS), AFC-S (AFEX-CS), EAC-S (EA-CS) and HYL (Hydrolysate).

**FIGURE 6:**
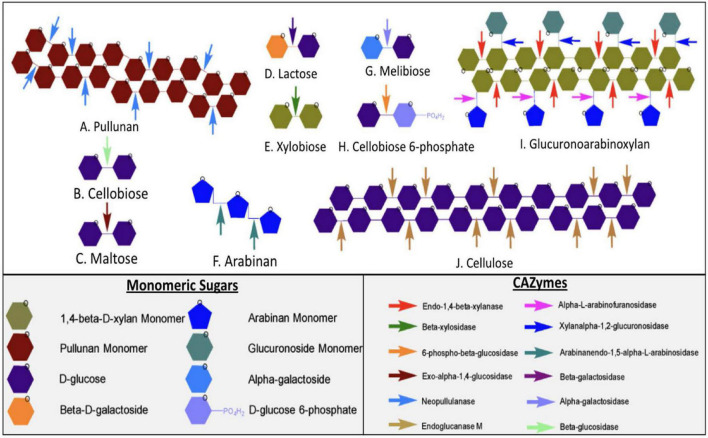
The different active sites of CAZymes and synergy between different enzymes present in *Geobacillus* sp. WSUCF1. At the top are the different types of sugar complexes represented by hexagons. The arrows are the CAZymes that breakdown different sugar linkages. The sugars are made up of their constituent monomeric sugars. Enzymes that are known to break down complex carbohydrate and dimeric sugars include A, Pullunan; B, Cellobiose; C, Maltose; D, Lactose; E, Xylobiose; F, Arabinan; G, Melibiose; H, Cellobiose 6-phosphate; I, Glucuronoarbinoxylan and J. cellulose.

Furthermore, some enzymes such as endoglucanase M were expressed consistently in all substrates except for Avicel. Additionally, some enzymes had similar expression patterns, as seen in Figure 4. GH family enzyme composition in secretomes produced using different substrates is given in Figure 5. From the figure, the GH52 enzyme occurred consistently in all substrate secretomes and was found to be more prominent when xylan was used to produce secretomes. Different enzymes present in *Geobacillus* WSUCF1 that break down complex and straightforward carbohydrate sugar linkages are given in Figure 6. AVI-S had the lowest variety in GH families with GH52, GH4, GH1, and GH13. Based on activity assay data and proteomics profile, it was observed that two secretomes (AVI-S and AFC-S) have high cellulase and xylanase activities, respectively. These two secretomes were selected to determine their hydrolytic efficiencies on AFEX-CS and EA-CS pretreated substrates.

### Synergistic Effect of WSUCF1 Secretome When Combined With Commercial Enzymes

**FIGURE 7:**
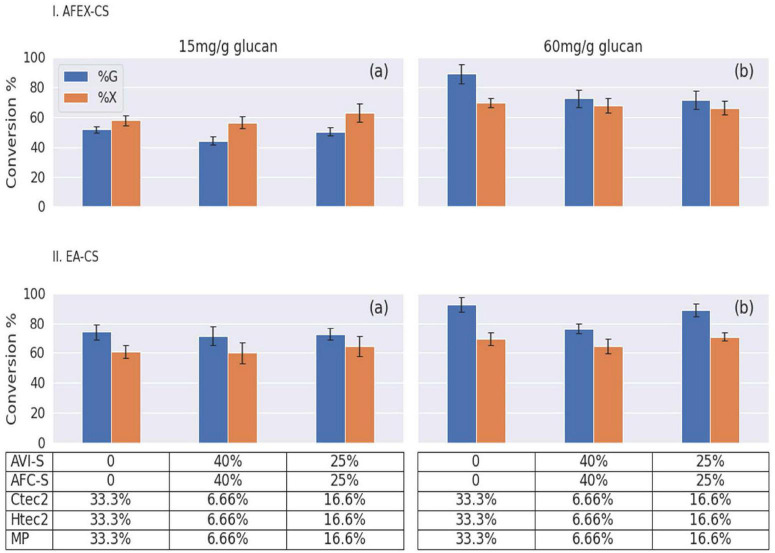
Bar graphs showing the percentage of glucan (G) and xylan (X) conversions for different pretreated corn stover after 24 h hydrolysis. Here, (I) AFEX-CS and (II) EA-CS were used as substrates. An equal amount of *Geobacillus* sp. WSUCF1 bacterial strain secretomes (AVI-S and AFC-S) and commercial enzymes (Ctec2, Htec2, and MP) at two enzymes loading [(a) 15 mg/g or (b) 60 mg/g of glucan] at two ratios (50:50 or 80:20). The x-axis on the graphs shows the percent of glucan/xylan conversion and y-axis show the different combinations of secretome produced using substrates given in the bracket. Here, AVI-S (Avicel), AFC-S (AFEX-CS); commercial enzymes (Ctec2, Htec2, and MP). On the y-axis the table shows the percentage of each enzyme mixture used.

Conversion of pretreated biomass requires many different enzyme activities to hydrolyze the sugar polymers to monomeric sugars. We designed experiments using different enzyme loadings and combinations of three different commercial enzymes (Ctec2, Htec2, and MP) and two secretomes (AVI-S and AFC-S) to hydrolyze pretreated biomass (AFEX-CS and EA-CS). Enzyme hydrolysis was carried out in a deep-well microplate for 24 h based on a previously established protocol using ground AFEX-CS and EA-CS as substrates (Chundawat et al., 2008). Secretomes and commercial enzymes were combined at two ratios (50:50 and 80:20) and two enzymes loading (low: 15 mg/g glucan and high: 60 mg/g glucan). Control experiments were done using 100% of commercial enzymes at both low- and high-enzyme loadings (Figure 7 and Supplementary Figures 1–4). In almost all the cases, EA-CS gave a higher glucan conversion when compared to AFEX-CS at low-enzyme loadings (15 mg/g of glucan). This is due to the removal of 44% of recalcitrant lignin and the formation of cellulose III (allomorph of cellulose, which is two times more reactive than native cellulose I) in corn stover during EA pretreatment as reported before (Chundawat et al., 2011c; da Costa Sousa et al., 2016a; Jin et al., 2016).

Interestingly, when 50% of commercial enzyme was replaced with an equal amount of secretomes (AVI-2 and AFC-S), we saw similar glucan and xylan conversion when compared to using 100% commercial enzymes in both AFEX-CS and EA-CS showing enzyme synergy. Producing two different secretomes using different substrates and mixing them for hydrolysis will be more expensive. To overcome this problem, we could produce the secretomes with better enzyme activities using mixed substrates (AFEX-CS and Avicel). When 80% of commercial enzymes were replaced with secretomes (AVI-S and AFC-S) in an equal ratio, we saw similar glucan, but slightly higher xylan conversion. When 100% of commercial enzyme loading was increased from 15 to 60 mg/g of glucan, we observed a 35% increase in glucan and a 10% increase in xylan conversions in AFEX-CS, and a 15% increase in glucan and 8% increase in xylan conversions in EA-CS. A similar trend was observed for 50:50 and 80:20 of commercial enzymes and secretome mixture for AFEX-CS, and only a marginal increase in glucan and xylan conversion was observed when using EA-CS. Displacing the commercial enzymes with 50–80% secretomes resulted in a 10% and 5% increase of glucan and xylan conversion for AFEX-CS and EA-CS, respectively, at low-enzyme loadings. However, no further increase in glucan and xylan conversion was observed at high-enzyme loadings. The secretome addition to the enzyme cocktail slightly improved the glucan and xylan conversion for AFEX-CS and EA-CS at low-enzyme loadings. However, at high-enzyme loading, commercial enzymes gave higher glucan and xylan conversion when compared to enzyme cocktails containing secretome.

### Sugar Conversion Efficiency of Secretome and Doping With Purified Fungal Cellobiohydrolases (CBHI and CBHII)

Two secretomes, namely AVI-S and AFC-S that gave the highest Avicelase and CMCase activities were chosen to test their sugar conversion efficiency on AFEX-CS and EA-CS at low- (15 mg/g of glucan) and high- (60 mg/g of glucan) enzyme loadings. As expected, high-enzyme loading gave higher glucan and xylan conversion, and EA-CS gave higher sugar conversion when compared to AFEX-CS. EA-CS at low-enzyme loading using 100% of AVI-S gave <15% glucan and 15% xylan conversion, respectively. On the other hand, using 100% of AFC-S gave <5% glucan and 45% xylan conversion. When AVI-S and AFC-S were combined in equal amounts, we just saw additive effects. We observed a significant improvement in sugar conversion (60% glucan and 50% xylan) when 90% AVI-S or AFC-S was combined with 10% of three commercial enzymes in an equal ratio. A similar higher sugar conversion was noticed when 90% of AFC-S was combined with 10% Ctec2 enzymes (Supplementary Figure 1). On the other hand, EA-CS at high-enzyme loading using 90% AVI-S and 10% Ctec2 or 10% MP or 10% equal mixture of Ctec2, Htec2, and MP gave >60% glucan and xylan conversion. A similar conversion was observed when 90% AFC-S was combined with a 10% equal mixture of Ctec2, Htec2, and MP enzymes (Supplementary Figure 2). These results show that AFC-S and AVI-S secretomes contained highly active hemicellulases and laccases that exhibited a high degree of synergism when combined with commercial fungal cellulases to increase the conversions by many folds. AFC-S secretome had a high potential for practical xylan conversions and did not require any commercial xylanases to get higher hemicellulose conversion.

We also evaluated the synergistic effects of adding purified CBHI and CBHII when combined with secretomes (AVI-S and AFC-S) on the hydrolysis of AFEX-CS and EA-CS at high (15 mg/g of glucan) and low (60 mg/g of glucan) enzyme loadings (Supplementary Figures 3, 4). At high-enzyme loading, adding 20% CBHI with 80% of AVI-S when hydrolyzing EA-CS gave 25% higher glucan conversion (50–75%) when compared to using 100% AVI-S. However, adding 20% CBHII enzyme with 80% AVI-S gave slightly higher glucan conversion when compared to CBHI under similar hydrolysis conditions. When 40% AVI-S and 40% AFC-S were combined with 10% CBHI and 10% CBHII, we observed a 76% glucan and 70% xylan conversion. Similar conversion results were seen when 40% AVI-S and 40% AFC-S were combined with 20% of commercial enzymes mixture (Ctec2, Htec2, and MP). These doping experiments clearly show that AVI-S and AFC-S secretomes lack CBHI and CBHII activity. Genetically modifying the *Geobacillus* WSUCF1 strain with genes harboring CBHI and CBHII enzymes may help to produce enzymes that can efficiently hydrolyze pretreated biomass to get higher sugar conversion. However, bacteria will not be able to properly glycosylate CBHI and II enzymes which are essential for their biological activity.

Various studies have been reported on the post-genomic analysis of microbes when fed on different biomasses. Proteomic profiles have been reported for various fungal species, e.g., *Phanerochaete chrysosporium* (Hori et al., 2011), *Postia placenta* (Martinez et al., 2009), *Ustilagomaydis* (Couturier et al., 2012), *Fusarium solani* (Scully et al., 2012), and *Irpexlacteus* (Salvachúa et al., 2013). There are very few reports on bacterial proteomes for lignocellulose degradation [*Clostridium phytofermentans* (Rydzak et al., 2012), *Clostridium thermocellum* (Tolonen et al., 2011), and *Thermobifida Fusca* (Adav et al., 2012a,b)]. In this study, the genome and proteome of *Geobacillus* WSUCF1 strain were studied. Analysis of the WSUCF1 strain genome elucidated a repertoire of genes for lignocellulose degradation. Various pure substrates and differently pretreated lignocellulosic biomasses were used for the investigation of biochemical response by WSUCF1 strain to produce secretome-containing biomass depolymerizing enzymes. Comparison of genome and secretomes of *Ustilago maydis* has been reported (Couturier et al., 2012). The WSUCF1 strain genome showed a more significant number of genes for hemicellulose hydrolysis as compared to cellulose hydrolysis. The WSUCF1 strain genome completely lacks genes for cellulose-degrading enzymes, CBHI and CBHII.

In the current study, we observed that secretomes AVI-S and AFC-S produced maximum active enzymes, which confirms that the enzyme expression in *Geobacillus* WSUCF1 is under the influence of inducible promoters. These promoters are induced particularly in the presence of soluble oligosaccharides produced from xylan and cellulose. Similar results of the inducible promoter were reported in the case of the well-known fungus *Daldinia decipiens oita* and *T. reesei* when grown on different carbon sources (Novy et al., 2019; Hori et al., 2020). Surprisingly, the genomic analysis showed that *Daldinia decipiens oita* lacks the lignin-degrading enzymes (Hori et al., 2020). Proteomic analysis revealed that a wide range of the CAZymes was expressed more in the production media that contained cellulose, poplar, and larch. However, when the media containing only glucose as the carbon source, the least number of enzymes were expressed. The *Geobacillus* WSUCF1 has the well-mechanized group of lignin-modifying enzymes, and recently Laccase (∼30 kDa) gene was cloned and expressed in *E. coli* through pRham N-His SUMO expression system (Rai et al., 2019). The lignocellulose deconstruction in the substrate takes place with the help of free radicals and reactive intermediates, resulting in stable and less toxic components essential for bacterial cell survival. Genome analysis of *Geobacillus* WSUCF1 reveals different genes that may express under stress conditions and could have played a key role in detoxification mechanisms. Some of the detoxifying enzymes include glutathione peroxidase, cytochrome c peroxidase, other peroxidases, and laccases (Table 2). Similar stress combatting enzymes were reported during the genomic and proteomic analysis of β-proteobacterium *Pandoraea* sp. ISTKB (Kumar et al., 2018). *Cytophaga hutchinsonii* lacks CHBI and exoglucanases fused to cellulose-binding domains (Xie et al., 2007). It has been reported that gene encoding cellulose-binding domains, CBHI and CBHII, were absent in cellulose-degrading fungus *Postia placenta* (Martinez et al., 2009). Two β-1,4 endoglucanase found in *Postia placenta* genome are similar to genes found in *Geobacillus* WSUCF1 strain.

*Geobacillus* WSUCF1 strain lacks crystalline cellulose-degrading enzymes such as CBHI and CBHII. Few other enzymes that have crystalline cellulose-degrading properties are present in *Geobacillus* WSUCF1 strain to overcome this deficiency. They include two genes for endoglucanase-M (EC 3.2.1.4), one gene for β-glucosidase (EC 3.2.1.21), one gene for 6-phospho-β-glucosidase (EC 3.2.1.86), and one gene for exo-α-1,4-glucosidase (EC 3.2.1.20). These enzymes can act synergistically to degrade the microcrystalline cellulose with exoglucanases that may possess the activity that enables it to liberate the cellobiose, which is then hydrolyzed by β-glucosidases to liberate glucose. Secretome data also confirmed this assumption, where all the above-mentioned cellulase activities were detected in proteome analysis. Martinez also suggested this mechanism for *Postia placenta*, which did not express any CBHI and CBHII in a cellulose-containing growth medium (Martinez et al., 2009). The presence of other GHs, i.e., 6-phospho-β-glucosidase and exo-α-1,4-glucosidase, expressed in secretomes are shown to produce glucose (Yu et al., 2013; Singh et al., 2016). Contrary to the WSUCF1 strain, proteomic analysis of *Clostridium thermocellum* showed the presence of cellulosomal complex for cellulose hydrolysis (Rydzak et al., 2012).

The WSUCF1 strain genome and proteome analysis also showed a complete set of enzymes for hemicellulose degradation (Table 2). Like the WSUCF1 strain, secretomes of *Clostridium thermocellum* and *Clostridium phytofermentans* on xylan showed the presence of endo-xylanases and β-xylosidase (Tolonen et al., 2011; Hirano et al., 2016). Thermophilic bacteria belong to the genus *Geobacillus*, are less explored for their lignocellulose degrading bio-catalytic systems, and are poorly understood because of lack of genetic evidence. The present genomic and proteomic analyses of *Geobacillus* WSUCF1 will help to fill the gap. Several cost-effective methods of producing novel enzymes from different microbial sources to saccharify the lignocellulosic biomass for producing fuels and chemical has been summarized (Tiwari et al., 2017). However, very few thermophilic enzymes are reported in the literature (Jørgensen et al., 2007; Bhalla et al., 2013a; Singh et al., 2019; Kaur et al., 2020), and that too by thermophilic bacterium (Novik et al., 2018; Kamble et al., 2019; Bajar et al., 2020). Some of the thermophilic CAZymes produced by *Geobacillus* WSUCF1 are reported to have superior enzyme stability and comparable enzyme activities to fungal enzymes (Rastogi et al., 2010; Bhalla et al., 2014, 2015; Rathinam et al., 2020). The *Geobacillus* sp. is a genus that contains twenty different bacterial species, and most of them are recognized for their obligatory thermophilic bacteria that are an excellent source for biotechnological bioprospecting.

Various hydrolytic enzymes are required to achieve efficient biodegradation of lignocellulosic biomass because of its inherent complexity and heterogeneity (Zambare and Christopher, 2012). *T. reesei* was developed to enhance cellulase production by overexpressing CBHII. An optimal enzyme cocktail was produced using a novel inducer mixture with soluble synthesized glucose-sophorose and alkali pretreated corn stover extracts (Li et al., 2017). When compared with centralized enzyme production, on-site enzyme production offers several cost-effective solutions for the saccharification of lignocellulosic biomass. However, dedicated efforts for practical implementation of the technology are needed to understand the real benefits. The number of enzymes produced (g/l) and the enzyme activities influence the economic efficiency of the process. In this study, we have demonstrated that *Geobacillus* WSUCF1 can produce highly active CAZymes using inexpensive pretreated corn stover as the substrate. We evaluated several substrates as a sole carbon source. Among them, secretomes produced using Avicel and AFEX-CS (AVI-S and AFC-S) gave the highest cellulase and xylanase activities, respectively. These two secretomes were selected for evaluating the hydrolytic efficiencies on AFEX-CS and EA-CS pretreated substrates. One possible reason for higher hemicellulase secretion using AFEX-CS could be the presence of water-soluble xylo-oligosaccharides produced during the pretreatment process (Chundawat et al., 2010, 2011a). In general, most of the lignocellulose degrading enzymes are under the control of inducible promoters (Singh and Arya, 2019) when oligosaccharides are present in the media.

The saccharification of lignocellulosic biomass requires different enzymes that act synergistically to produce glucose and xylose. Secretome AFC-S efficiently converted the xylan component of AFEX-CS and EA-CS into xylose, and the conversions were comparable to the conversions obtained with commercial enzymes. In addition to endo-1, 4-β-xylanase in AFC-S, other accessory enzymes such as β-xylosidase, xylan-α-1,2-glucuronidase, α-L-arabinofuranosidase, and arabinanendo-1,5-α-L-arabinosidase helped in converting biomass with high xylose yields. Gao et al. (2011) reported a sixfold increase in the xylose yields with the addition of β-xylosidase, xylan-α-1, 2-glucuronidase, and α-L-arabinofuranosidase as accessory enzymes to endo-1,4-β-xylanase. When adding AFC-S and AVI-S secretomes to AFEX-CS or EA-CS, the glucose yields were lower compared to commercial enzymes. Doping of CBHI and CBHII to the AVI-S secretome helped increase the sugar yields. However, doping CBHI produced higher glucose yields when compared to using CBHII. den Haan et al. (2013) also reported higher glucose yields of 16.5% with CBHI doping compared to 9.9% achieved with CBHII doping at the same enzyme loading. An increase in the glucan conversions with CBHI and CBHII enzymes mixed with AVI-S and AFC-S secretomes showed the high synergy between the enzymes from bacteria and fungi (Selig et al., 2008). Synergism studies between cellulases and xylanases for enhanced lignocellulose conversion had been reported previously (Gao et al., 2010a,b, 2011). Wang et al. (2019) reported the synergy operating between xylanolytic *Bacillus* strain in the presence of commercially available cellulase of *T. reesei* using ammonia-pretreated corn stover as a feedstock at pH 6.0 and 65°C. Most microbes are either cellulolytic or hemicellulolytic, depending on the environment where they survive. It is sporadic to find an organism that produces both cellulase and hemicellulase for efficient saccharification of biomass biocatalysts for the lignocellulose saccharification (Cortes-Tolalpa et al., 2017). Our studies reconfirm the results of the previous report that enzymes secreted by *Geobacillus* WSUCF1 have hemicellulase activities (Bhalla et al., 2013a; Dragone et al., 2020). Also, the results from these studies show that enzymes produced by *Geobacillus* WSUCF1 synergize with commercial fungal enzymes during saccharification of lignocellulosic biomass, which could help to reduce the saccharification cost when produced on-site in a biorefinery.

## Conclusion

The detection of bacterial enzymes that display novel activities is essential to efficiently break down lignocellulosic biomass to produce biofuels. The proteomic profiles revealed the potential of *Geobacillus* WSUCF1 as an efficient degrader of lignocellulose with key CAZymes. Genomic data for hydrolyzing enzymes matched with the post-genomic data of the isolate. A cocktail of xylanases and accessory enzymes was identified in the AFC-S secretome. Doping of the CBHI and CBHII enzymes or 10% of commercial cellulase to AFC-S and AVI-S secretomes effectively hydrolyzed EA-CS, resulting in sugar conversion similar to commercial enzymes alone. Our study gave new insights into the biomass conversion processes.

## Data Availability Statement

The datasets presented in this study can be found in online repositories. The names of the repository/repositories and accession number(s) can be found below: PRIDE Archive - PXD031733.

## Author Contributions

VB and AB came with the concept, identified the team members to work on this project, collectively designed the experiments, and contributed to the manuscript writing. AB did most of the lab experiments. VB helped to interpret the data, secure the funds from Great Lakes Bioenergy Research Center (GLBRC). JA did the *Geobacillus* WSUCF1 genome annotation and proteomic analysis and contributed to writing the manuscript. BU helped to draft some of the figures in the manuscript. GS helped in interpreting the hydrolysis experimental data and contributed to the manuscript wrote-up. RS provided the *Geobacillus* WSUCF1 strain and helped in interpreting the genome annotation data and contributed to manuscript correction. All authors contributed to the article and approved the submitted version.

## Conflict of Interest

The authors declare that the research was conducted in the absence of any commercial or financial relationships that could be construed as a potential conflict of interest.

## Publisher’s Note

All claims expressed in this article are solely those of the authors and do not necessarily represent those of their affiliated organizations, or those of the publisher, the editors and the reviewers. Any product that may be evaluated in this article, or claim that may be made by its manufacturer, is not guaranteed or endorsed by the publisher.

## Footnotes

1
www.thermofisher.com

2
www.matrixscience.com

3
www.proteomesoftware.com
