# Supplementary material for: Thermophilic Geobacillus WSUCF1 Secretome for Saccharification of Ammonia Fiber Expansion and Extractive Ammonia Pretreated Corn Stover
Source: Front Microbiol. 2022 May 25;13:844287. doi: 10.3389/fmicb.2022.844287 (PMC9176393; doi:10.3389/fmicb.2022.844287)
Supplement: Supplementary file 1 [file Data_Sheet_1.pdf]

Supplementary Figures:

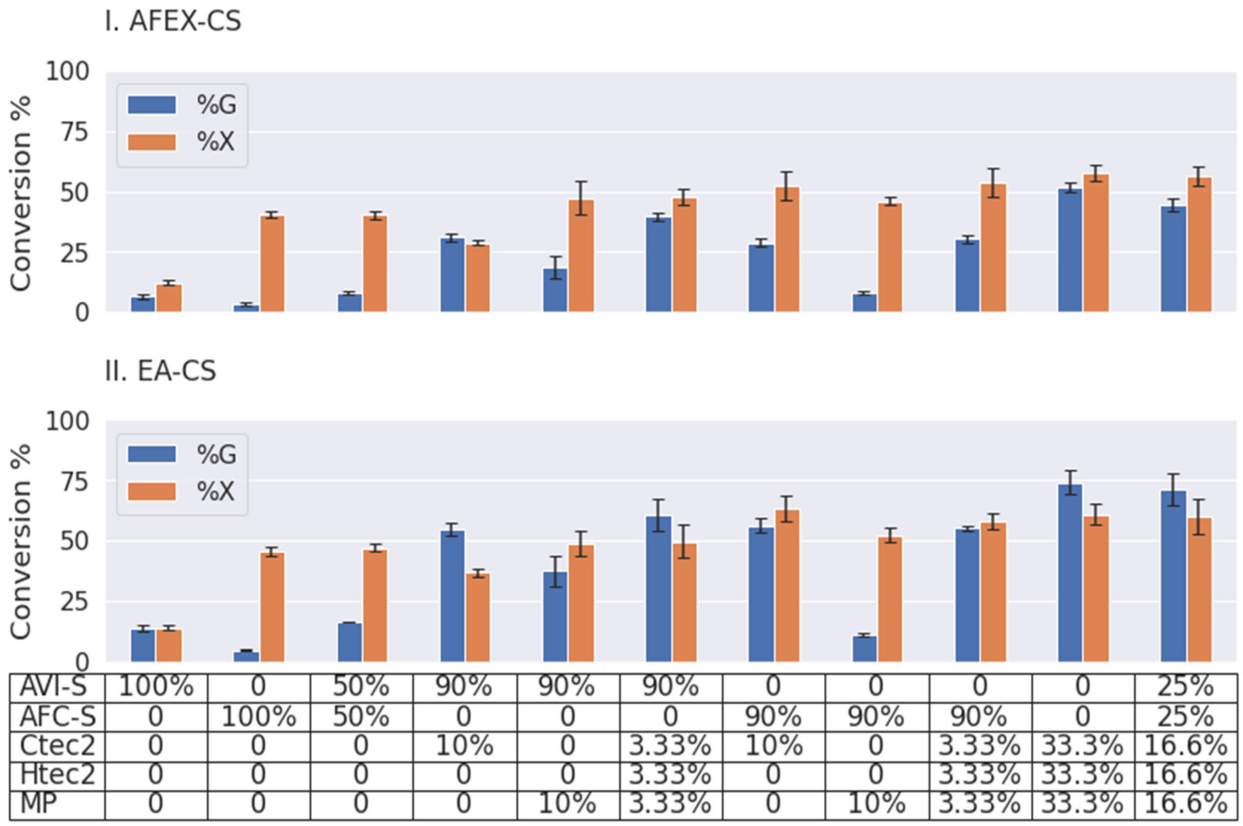

AVI-S	100%	0	50%	90%	90%	90%	0	0	0	0	25%
AFC-S	0	100%	50%	0	0	0	90%	90%	90%	0	25%
Ctec2	0	0	0	10%	0	3.33%	10%	0	3.33%	33.3%	16.6%
Htec2	0	0	0	0	0	3.33%	0	0	3.33%	33.3%	16.6%
MP	0	0	0	0	10%	3.33%	0	10%	3.33%	33.3%	16.6%

Figure S1. Bar graphs showing the glucose and xylose percent conversions for different ammonia pretreated corn stover. Here, (I) AFEX-CS and (II) EA-CS substrates used for hydrolysis with a total enzyme loading of 15 mg/g glucan in 24 h. A combination of *Geobacillus* sp. WSUCF1 bacterial strain secretomes and commercial enzymes were mixed and used for xylose and glucose conversions. The x-axis on the graphs shows the percent of glucose/xylose conversion and y-axis show the different combinations of secretome produced using substrates given in the bracket, AVI-S (Avicel), AFC-S (AFEX-CS) and commercial enzymes (Ctec2, Htec2, and MP). On the y-axis the table shows the percentage of each enzyme mixture used.

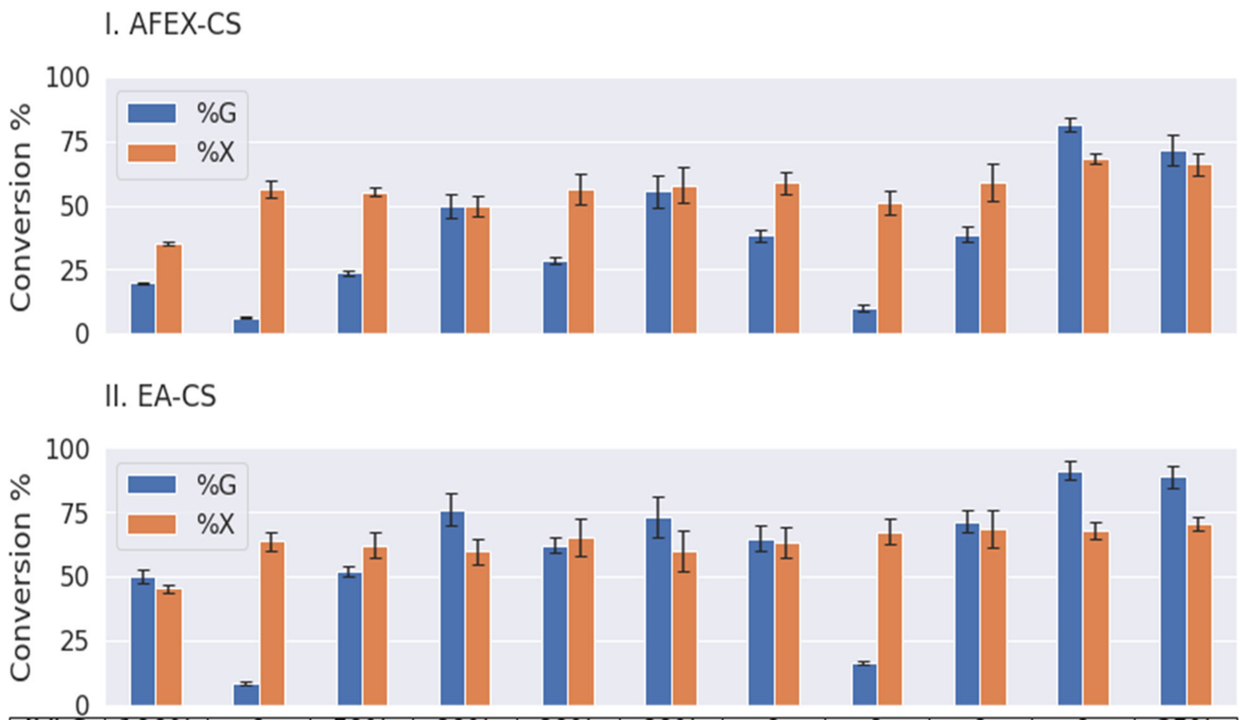

AVI-S	100%	0	50%	90%	90%	90%	0	0	0	0	25%
AFC-S	0	100%	50%	0	0	0	90%	90%	90%	0	25%
Ctec2	0	0	0	10%	0	3.33%	10%	0	3.33%	33.3%	16.6%
Htec2	0	0	0	0	0	3.33%	0	0	3.33%	33.3%	16.6%
MP	0	0	0	0	10%	3.33%	0	10%	3.33%	33.3%	16.6%

Figure S2. Bar graphs showing the glucose and xylose percent conversions for different ammonia pretreated corn stover. Here, (I) AFEX-CS and (II) EA-CS substrates used for hydrolysis with a total enzyme loading of 60 mg/g glucan in 24 h. A combination of *Geobacillus* sp. WSUCF1 bacterial strain secretomes and commercial enzymes were mixed and used for xylose and glucose conversions. The x-axis on the graphs shows the percent of glucose/xylose conversion and y-axis show the different combinations of secretome produced using substrates given in the bracket, AVI-S (Avicel), AFC-S (AFEX-CS) and commercial enzymes (Ctec2, Htec2, and MP). On the y-axis the table shows the percentage of each enzyme mixture used.

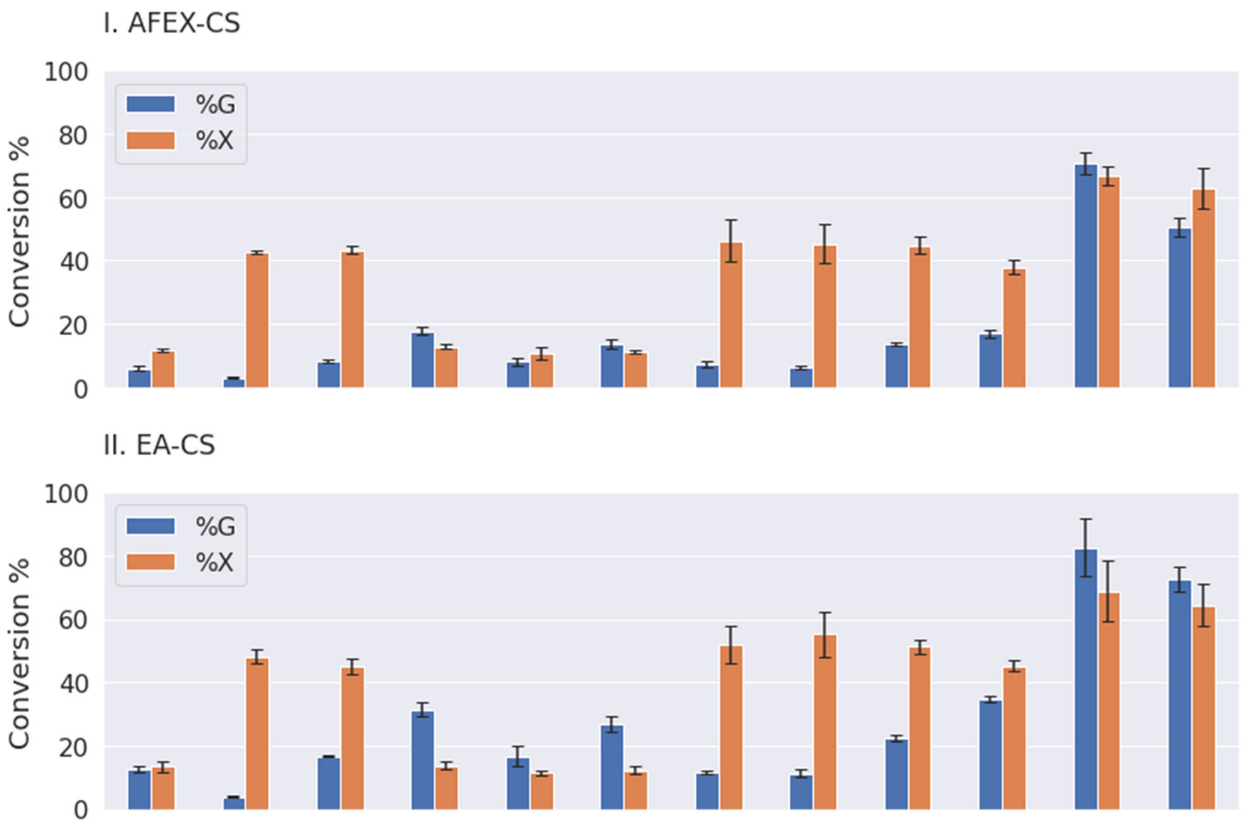

AVI-S	100%	0	50%	80%	80%	80%	0	0	0	40%	0	40%
AFC-S	0	100%	50%	0	0	0	80%	80%	80%	40%	0	40%
Ctec2	0	0	0	0	0	0	0	0	0	0	33.3%	6.66%
Htec2	0	0	0	0	0	0	0	0	0	0	33.3%	6.66%
MP	0	0	0	0	0	0	0	0	0	0	33.3%	6.66%
CBHI	0	0	0	20%	0	10%	20%	0	10%	10%	0	0
CBHII	0	0	0	0	20%	10%	0	20%	10%	10%	0	0

Figure S3. Bar graphs showing the glucose and xylose percent conversions for different ammonia pretreated corn stover. Here, (I) AFEX-CS and (II) EA-CS substrates used for hydrolysis with a total enzyme loading of 15 mg/g glucan in 24 h. A combination of *Geobacillus* sp. WSUCF1 bacterial strain secretomes and commercial enzymes were mixed and used for xylose and glucose conversions. The x-axis on the graphs shows the percent of glucose/xylose conversion and y-axis show the different combinations of secretome produced using substrates given in the bracket, AVI-S (Avicel), AFC-S (AFEX-CS); commercial enzymes (Ctec2, Htec2, and MP) and purified enzymes (CcBHI and CcBHII). On the y-axis the table shows the percentage of each enzyme mixture used.

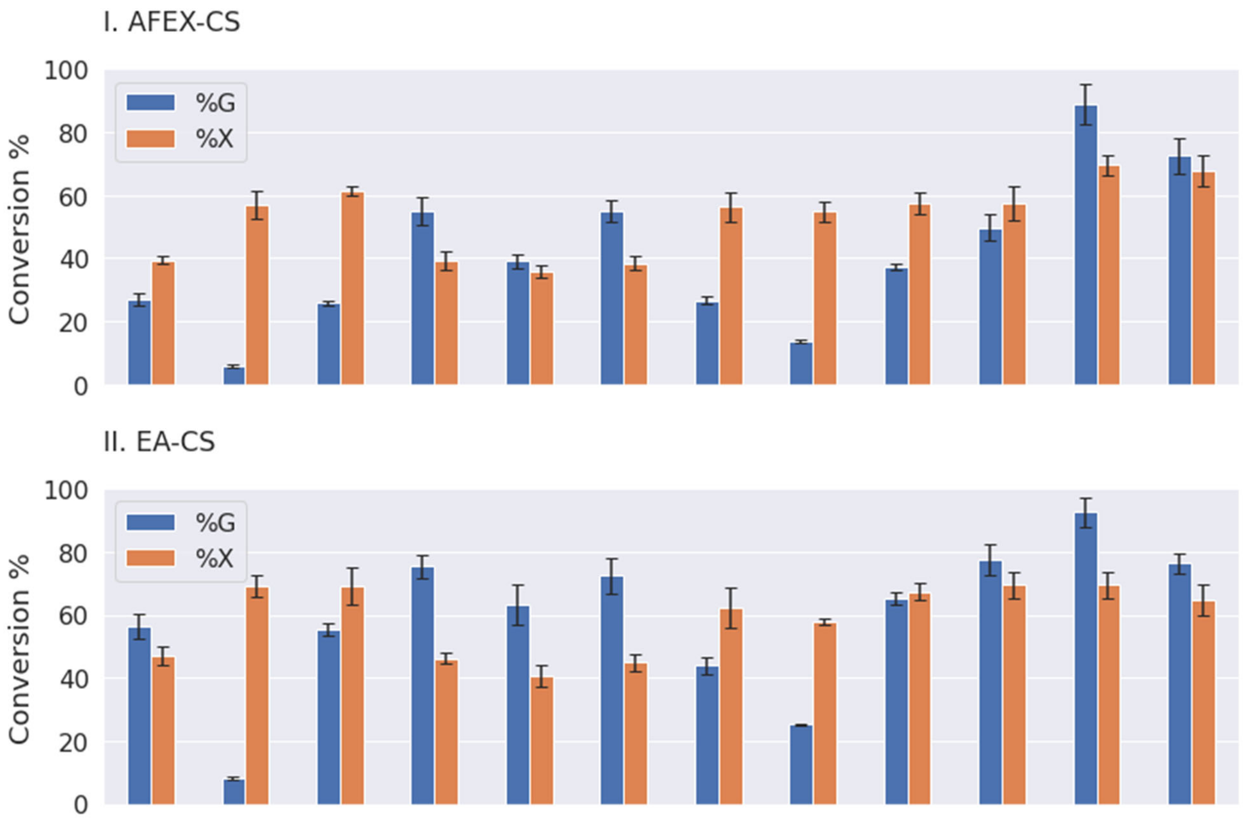

AVI-S	100%	0	50%	80%	80%	80%	0	0	0	40%	0	40%
AFC-S	0	100%	50%	0	0	0	80%	80%	80%	40%	0	40%
Ctec2	0	0	0	0	0	0	0	0	0	0	33.3%	6.66%
Htec2	0	0	0	0	0	0	0	0	0	0	33.3%	6.66%
MP	0	0	0	0	0	0	0	0	0	0	33.3%	6.66%
CBHI	0	0	0	20%	0	10%	20%	0	10%	10%	0	0
CBHII	0	0	0	0	20%	10%	0	20%	10%	10%	0	0

Figure S4. Bar graphs showing the glucose and xylose percent conversions for different ammonia pretreated corn stover. Here, (I) AFEX-CS and (II) EA-CS substrates used for hydrolysis with a total enzyme loading 60 mg/g glucan in 24 h. A combination of *Geobacillus* sp. WSUCF1 bacterial strain secretomes and commercial enzymes were mixed and used for xylose and glucose conversions. The x-axis on the graphs shows the percent of glucose/xylose conversion and y-axis show the different combinations of secretome produced using substrates given in the bracket, AVI-S (Avicel), AFC-S (AFEX-CS); commercial enzymes (Ctec2, Htec2, and MP) and two purified fungal cellobiohydrolases (CBHI and CBHII). On the y-axis the table shows the percentage of each enzyme mixture used.
